# Clinical evaluation of a motion correction software based on partial angle reconstruction in coronary CT angiography

**DOI:** 10.1007/s10554-026-03678-w

**Published:** 2026-03-09

**Authors:** Marco Caballo, Joanne D Schuijf, Matthew Benbow, Andrea Foden, Laura McLennan, Mark Condron, Sue Thomas, Russell Bull

**Affiliations:** 1 Computed Tomography, Canon Medical Systems Europe, Bovenkerkerweg 59, Amstelveen, 1185 XB the Netherlands; 2 Global RDC, Canon Medical Systems Europe, Bovenkerkerweg 59, Amstelveen, 1185 XB the Netherlands; 3 CT & MRI Department, Royal Bournemouth Hospital, University Hospitals Dorset, Castle Lane East, Bournemouth, BH7 7DW UK; 4 Computed Tomography, Canon Medical Systems UK, Boundary Court, Gatwick Road, Crawley, RH10 9AX UK

**Keywords:** coronary CT angiography, wide-area detector, motion correction, deep learning, partial angle reconstruction

## Abstract

To evaluate a new deep learning (DL) motion correction (MC) software based on partial angle reconstruction (PAR) to reduce motion artifacts in patients with increased heart rate (HR) in coronary CT angiography (CCTA). This retrospective single-center study included consecutive patients with HR > 70 bpm who underwent single-beat wide-area-detector CCTA over a 6-month period. A DL PAR-based MC software was applied to each image, and corrected and uncorrected reconstructions were scored by two blinded independent cardiothoracic radiologists for coronary motion artifact severity. Scores were obtained on a per-vessel and on a per-patient level using a 5-point Likert scale (1 = non-interpretable, 2 = severe, 3 = moderate, 4 = mild, 5 = no artifacts). Scoring differences were analyzed with Chi-Squared test and interrater agreement with Gwet agreement coefficients. 62 patients (35 female) with (mean ± std.dev.) BMI 29.4 ± 6.8 kg/m2 and HR 81.9 ± 13.1 bpm were included. Without MC, the number of cases scored 3 or higher on a per-patient level were 40/62 (64.5%) and 43/62 (69.4%), respectively for reader 1 and 2. With MC, they improved to 50/62 (80.6%) and 55/62 (88.7%), respectively for reader 1 and 2. Improvements in scoring were significant for both readers (*p* < 0.02). Per-vessel scores followed a similar trend, but showed significance for both readers only for the right coronary artery (*p* < 0.001). The fraction of diagnostically-interpretable cases (score ≥ 2) were 91.9% (uncorrected) and 98.4% (motion-corrected) (reader 1), and 93.5% (uncorrected) and 96.8% (motion-corrected) (reader 2). Interrater agreement was between (0.67–0.78). The MC software significantly improved image quality by reducing coronary motion artifacts in CCTA patients with increased HR.

## Introduction

Non-invasive coronary computed tomography angiography (CCTA) has become the frontline clinical choice for the evaluation and follow-up of coronary artery diseases (CAD) [1]. Despite the substantial technological improvements and clinical benefits observed in the last few years, cardiac motion remains an important challenge that may hinder the diagnostic outcome of CCTA [2].

Different solutions are available to limit motion in CCTA. Dual-source CT reduces the acquisition angle to a quarter of the gantry rotation time, achieving a twofold increase in nominal temporal resolution compared to single-source CT. However, while possible to achieve near-whole-heart coverage in a single heartbeat under appropriate heart rate (HR) ranges or high-pitch acquisition modes, dual source technology often requires sequential or spiral acquisition in patients with elevated heart rate or irregular rhythm. Therefore, especially in patients with higher HR, imaging occurs over multiple beats, increasing the risk of temporal non-uniformity, misregistration (stair-step) artifacts, and potentially increasing dose [2–3]. Wide-area detector CT has sufficient coverage to image the heart in one rotation, but the nominal temporal resolution is limited to half the gantry rotation time. Therefore, in patients with high HR, multi-segment reconstruction may be needed to improve the effective temporal resolution. This carries the disadvantages of requiring multiple cardiac cycles, of being sensitive to arrhythmia, and of a potential increase in delivered radiation dose. Both dual source and wide-area detector CT may therefore require multi-beat imaging depending on patient heart rate, rhythm, and depending on scanner characteristics and acquisition mode. To mitigate the potential drawbacks of multi-beat imaging and expand one-beat acquisition to more patients, motion correction (MC) algorithms have been proposed, aimed at reducing possible residual artifacts deriving from cardiac motion in a reconstruction or postprocessing phase.

One of the most prominent approaches in cardiac motion correction is the estimation of a motion vector field (MVF) from multiple reconstructed phases of the cardiac cycle [4–9]. In these phase-based motion correction methods, the MVF is calculated by evaluating voxel-wise displacements between additional acquired phases, and is used to correct for motion in the target phase [10]. While promising, phase-based motion correction approaches may present some theoretical potential pitfalls. As they require multiple phases to estimate the MVF, the quality of this latter is theoretically dependent on the quality of the target cardiac phase and on the duration of the additional phases. Furthermore, and for the same reason, there might be a limit in reducing the acquisition window, and this could result in the potential suboptimal use of x-ray dose or in theoretical constraints in achieving a substantial dose reduction.

An alternative method more recently introduced is represented by partial angle reconstruction (PAR), which divides the scan into small angular segments and reconstructs them individually [11–12]. Although they suffer from limited-angle artifacts, these segments present a high temporal resolution. This allows for an accurate estimation of the MVF, which can be calculated through the application of deep learning (DL) algorithms [13–14]. While their potential is yet to be fully explored in clinical practice, PAR-based motion correction approaches present the theoretical advantage of being less dependent on the target acquisition phase, since they do not estimate the MVF directly in the reconstruction domain from multiple phases. Furthermore, thanks to the large number of projections derived from the fast gantry rotation time in modern CT scanners, the MVF can be calculated within a single heartbeat, potentially allowing to expand the indications of one-beat imaging to more patients. Therefore, the investigation of PAR-based approaches in clinical practice, while still at a preliminary stage, is of interest. Based on studies primarily with simulated data, PAR-based approaches showed high potential to improve CCTA image quality [12–13]. However, to the best of our knowledge, previous research describing their potential in the clinics, and especially in increased HR patients, is currently missing. Therefore, the purpose of this study was to evaluate the performance of a new DL PAR-based MC software in patients with increased HR acquired in single-beat wide-area detector CCTA.

## Materials and methods

### Patient population

This retrospective study was conducted in an acute general hospital (Royal Bournemouth Hospital, University Hospitals Dorset, Bournemouth, United Kingdom) in accordance with the guidelines of the Declaration of Helsinki. As the study involved retrospective images from patients acquired during the normal clinical routine, and as written informed consent for re-use of patient anonymized data for research purposes was obtained, the institutional review board (IRB) approval was not required.

Images of consecutive patients referred to CCTA for suspected CAD during a time period of 6 months (January 2025 - July 2025) were retrospectively selected. Exclusion criteria were patients referred to CT for TAVI planning and pre-ablation procedures, and patients with HR lower than 70 beats per minute (bpm) at the time of image acquisition. No additional exclusion criteria were adopted with respect to clinical history, clinical findings, or image quality. In other words, all consecutive patients referred to CCTA for suspected CAD with HR higher than 70 bpm were included. None of the patients analyzed in this study were reported in previous work.

### CT acquisition and reconstruction

Images were acquired following practice guidelines [15] on a clinical 320-row detector CT system with gantry rotation time 0.24 s (Aquilion ONE INSIGHT Edition, Canon Medical Systems, Otawara, Japan). All patients were scanned in volume mode in a single heartbeat with prospective ECG triggering. Tube voltage was selected based on automatic kV selection (^SURE^kV, limited to 100 kV or 120 kV for the CCTA protocol used in our routine), with collimation 120–160 mm, and tube current was automatically modulated with a target noise standard deviation of 38 Hounsfield units (^SURE^Exposure) calculated on a 3D ultra-low-dose localizer (3D Landmark Scan [16–17]). The planned exposure window was selected according to the patient HR and HR variability during breath exercise. The possible pre-set exposure windows were 73–77%, 70–80% and 30–80%. CT dose index-volume (CTDI-vol) and dose length product (DLP) were extracted from each examination, and the effective dose (ED) calculated (conversion factor 0.026 mSv.[mGy.cm]^−1^). For each patient, the following characteristics were recorded: age, gender, height, weight, body mass index (BMI), HR at acquisition and HR variability. As all acquisitions were performed in a single heartbeat, the HR variability could not be calculated during acquisition, and was therefore calculated from the breath exercise both as standard deviation from mean HR, and as range of HRs [18].

Patients were administered sublingual nitroglycerin and intravenous Metoprolol (up to 50 mg) unless contraindicated. Depending on the kV selection upon patient BMI, 70–100 ml of contrast agent (Omnipaque 350, 350 mg I/ml, GE Healthcare Limited) were injected at a flow rate of 4.5-6.0 ml/s, followed by 40–50 ml of saline solution. Acquisition was automatically triggered using bolus tracking performed on the 3D localizer at a threshold of 180 Hounsfield units. Images were reconstructed with the super-resolution deep learning reconstruction method Precise IQ Engine (PIQE Cardiac level 2, Canon Medical Systems) [19–20], with matrix size 1024 × 1024, with slice thickness and interval of 0.5 mm and 0.25 mm, respectively. Following normal clinical routine at our institution, all images were reconstructed with automatic best-phase selection and with the 75% phase. For each case, the best phase between that automatically selected by the scanner and the 75% phase was selected by a board-certified cardiothoracic radiologist.

### Motion correction

**Fig. 1:**
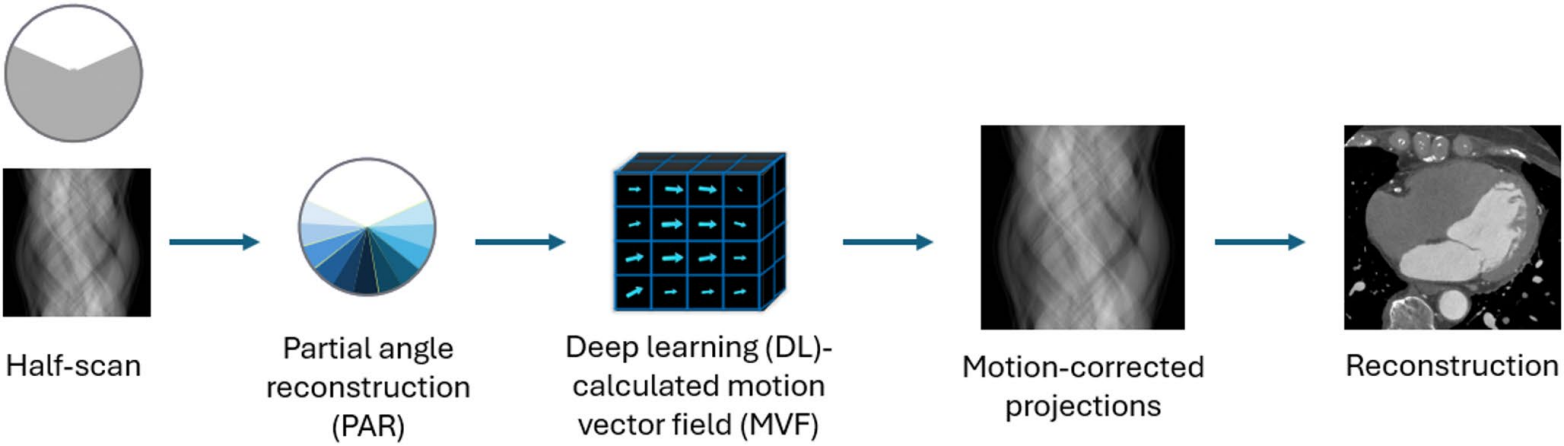
Illustration of the main steps of the motion correction software evaluated in this study

Each image was motion-corrected using a commercially available software (CLEAR Motion Cardiac, Canon Medical Systems) integrated on the CT system. The main working principle of the software is briefly described in this section. After an ECG-triggered half-scan acquisition, the software divides the projection data into multiple segments of 14 millisecond temporal resolution each. These segments undergo PAR to generate as many limited-angle images, each capturing the heart in a different temporal moment. These images are subsequently analyzed with a DL model based on deep convolutional neural networks, which estimates the MVF by analyzing the displacement of cardiac structures across these multiple segments. Once the MVF is calculated, it is applied during image reconstruction using the desired reconstruction method, resulting in motion-corrected images. The main steps of the MC software are shown in Fig. 1.

### Image quality scoring

Each uncorrected and motion-corrected image was scored by two independent board-certified cardiothoracic radiologists (ST and RB, 13 and 25 years of experience) using a 5-point Likert scale. Radiologists were asked to score on a per-vessel level for coronary artery motion artifacts (1 = non-interpretable findings, 2 = suboptimal image quality with severe artifacts, 3 = good image quality with moderate artifacts, 4 = very good image quality with mild artifacts, 5 = excellent image quality with no artifacts). A per-patient level score was determined by the lowest score assigned among the three coronary arteries, and a case was considered diagnostically interpretable on a patient level if all coronary arteries were scored with a 2 or higher. Readers were blinded to any image or patient information, and reading was performed in multiple batches with randomized image order and with a 4-week interval between batches to minimize recall bias. Uncorrected and motion-corrected images were mixed randomly with a 50%-50% ratio in the different sessions separated by the washout time interval. Each image was scored while being shown on a calibrated diagnostic display monitor in a radiology reading room with no time limit. Subjective image quality scoring between uncorrected and motion-corrected images was analyzed for all patients, and a sub-group analysis was performed with a cut-off value of 75 bpm.

### Correlation with patient and imaging characteristics

The effect of imaging and patient characteristics on differences in image quality scoring between motion-corrected and uncorrected images was evaluated through multivariate linear regression, for both readers. Imaging characteristics included CTDI-vol, DLP, and administered contrast medium volume and flow rate. Patient characteristics included weight, height, BMI, HR at acquisition, HR variability, and degree of coronary artery calcification. As calcium scoring is not routinely performed at our institution, the degree of coronary calcification was assessed for all images by a Health and Care Professions Council (HCPC) registered diagnostic radiographer with 24 years of experience. Each image was scored as either containing none (0), limited (1), or extensive (2) coronary artery calcification. This assessment was performed in a blinded fashion with respect to the radiologist scoring, and with images presented in random order.

### Objective image quality

To assess whether the MC software introduced modification in image noise and contrast, each uncorrected and motion-corrected image was analyzed quantitatively by a Health and Care Professions Council (HCPC) registered diagnostic radiographer with 24 years of experience. Circular regions of interest (ROIs) were placed in the ascending aorta at the level of the origin of the left main artery and in the surrounding perivascular adipose tissue, following previously published methodologies [18, 21]. Each ROI was placed on the motion-corrected image and copied to the respective uncorrected image in the same location. For each image, the average Hounsfield units (HU) of the aorta and adipose tissue, and the standard deviation (SD) of the aorta, were calculated. Image noise, signal-to-noise ratio (SNR) and contrast-to-noise ratio (CNR) were calculated as follows.

1 $$Noise={SD}_{aorta}$$

2 $$SNR={HU}_{aorta}/{SD}_{aorta}$$

3 $$CNR={(HU}_{aorta}-{HU}_{adipose})/{SD}_{aorta}$$

**Fig. 2:**
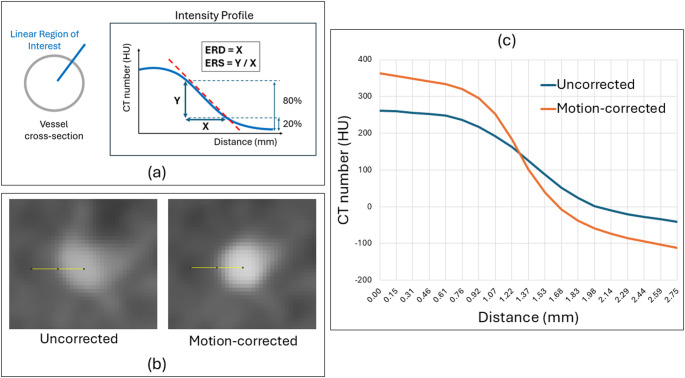
(**a**) Schematics of the calculation of the edge rise distance (ERD) and edge rise slope (ERS); (**b**) Examples of cross-sections of mid-points of the right coronary artery (RCA) from the same patient, without (left) and with motion correction (right); (**c**) extracted CT vessel profiles from the two RCA cross-sections (uncorrected and motion-corrected), used to calculate the ERD and ERS

In addition to noise, SNR, and CNR, an additional objective metric was used to quantify the degree of motion artifact reduction from uncorrected to motion-corrected images. For this, the edge rise distance (ERD) and the edge rise slope (ERS) were calculated from the CT attenuation profile of the right coronary artery. The profile was obtained by evaluating the HU numbers along a linear ROI placed over the cross-section of the middle point of the right coronary artery (typically the part of the vessel most prone to motion). The ROI was drawn, for each case, on the motion-corrected image and copied to the uncorrected one. Only a single point of a single vessel (mid-point of the right coronary artery) was evaluated, to quantify the motion correction effect in the location most subjected to motion, and to be aligned with the conservative criterion applied to our subjective analysis (i.e., patient level scoring as the worst of the scores among all vessels). A schematics and example of ERD and ERS calculation is shown in Fig. 2. A lower value of ERD and a higher value of ERS indicate a sharper vessel profile, thereby indicating a lower magnitude of motion artifacts. Measurements were performed twice per patient and the average ERD and ERS values were used for analysis.

Analyses on noise, SNR and CNR were performed using Vitrea Advanced Visualization (Version 7.16.0.933, Canon Medical Informatics, Inc, Minnetonka, MN, USA). Calculation of ERD and ERS were performed using ImageJ (NIH, Bethesda, Maryland, USA).

### Statistical Analysis

The normality of distributions of each variable was assessed by Shapiro-Wilk testing. Quantitative variables are expressed as mean (standard deviation), or median and interquartile range (IQR), as applicable. Categorical variables are reported as counts or percentages. T-test was used to compare normally distributed continuous variables. Wilcoxon test was used to compare ordinal continuous variables. Chi-squared test was used to compare distributions of categorical variables.

In the multivariate regression analysis performed to evaluate the potential correlation between differences in image quality scoring and patient and imaging characteristics, the influence of each variable on the measured outcome was assessed by evaluating statistical significance of the ratio of explained / unexplained variance (F-statistics) [22].

Interobserver agreement between the two radiologists was calculated for each image quality score with the Gwet agreement coefficient (linear weights). This metric was chosen over the more commonly used Cohen weighted kappa score as it was shown to be more robust even in case of little dispersion in scores, thus generally representing a more reliable measurement of the presence (or absence) of agreement [23–24, 25].

The results of the subjective image quality scoring were also analyzed using visual grading characteristics (VGC) [26–27], a non-parametric rank-invariant statistical method aimed at determining differences in image quality between two groups of images (in our case, uncorrected and motion-corrected reconstructions). The VGC analysis summarizes the differences in image quality scoring in a single curve (for both readers together) following the receiver operating characteristics (ROC) theory, with an area under the curve (AUC) statistically larger than 0.5 indicating significant differences between the two group distributions. This analysis was performed both at a patient and at a vessel level, and VGC curves, AUC, 95% confidence interval (CI), and p-value testing the difference from random distribution (AUC = 0.5) were reported.

Statistical significance was assumed in all cases at a two-tailed P value < 0.05. All analyses were performed in MATLAB (R2018a, The MathWorks, Natick, MA, USA), except for the VGC analysis, performed with an openly available previously validated software [28–29].

## Results

**Fig. 3:**
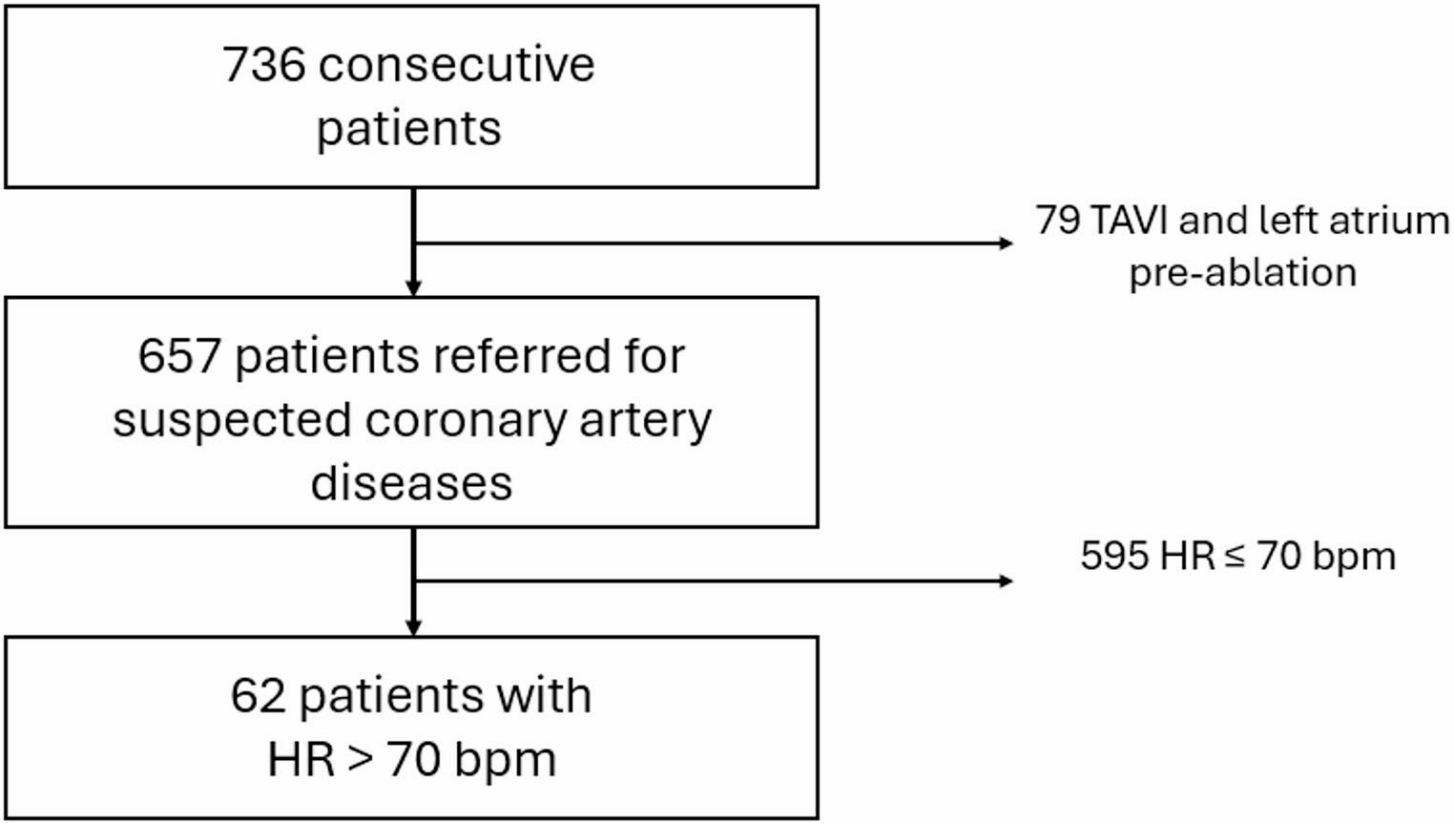
Flowchart of patient selection

A total of 736 patients were referred to CCTA in the time frame of this study. 79 patients were excluded as not referred for suspected coronary artery disease (TAVI, left atrium pre-ablation), and 595 patients were excluded due to their HR at acquisition being ≤ 70 bpm. As a result, 62 patients (35 female, 27 male) with HR > 70 bpm were included (flowchart of patient selection shown in Fig. 3). The majority of the included patients (60/62) were acquired at both end systole and diastole (30–80%), one at 70–80%, one at 73–77%.

All baseline characteristics are reported in Table 1. The average (SD) age was 64.9 (12.5) years, the BMI was 29.4 (6.8) kg/m^2^, and the HR at acquisition was 81.9 (13.1) bpm. The average CTDI-vol, DLP and ED were 18.3 (10.9) mGy, 239.9 (139.1) mGy.cm, and 6.2 (3.6) mSv. Of all patients included, the number of cases with HR (70 bpm − 75 bpm), [75 bpm – 80 bpm], and above 80 bpm were 25/62 (40%), 12/62 (20%), and 25/62 (40%) (respectively). The highest HR in our cohort was 123 bpm. The degree of coronary calcifications was scored as none, limited and extensive in 24 (39%), 29 (47%), and 9 (14%) patients, respectively.

**Table 1:** Baseline characteristics. Unless otherwise indicated, data are mean (standard deviation, SD)

Characteristic	Value (*n* = 62)
Female	35
Male	27
Age, years	64.9 (12.5)
Body weight, kg	85.5 (22.1)
Body height, m	1.7 (0.1)
Body mass index, kg/m^2^	29.4 (6.8)
Heart rate at acquisition, bpm	81.9 (13.1)
Heart rate variability (SD), bpm *	1.5 (0.5–6.9)
Heart rate variability (range), bpm **	3.0 (1.0–13.8)

*Heart rate variability calculated as standard deviation from mean heart rate. Data are median; data in parentheses are interquartile range

**Heart rate variability calculated as range of heart rates during data acquisition. Data are median; data in parentheses are interquartile range

**Fig. 4:**
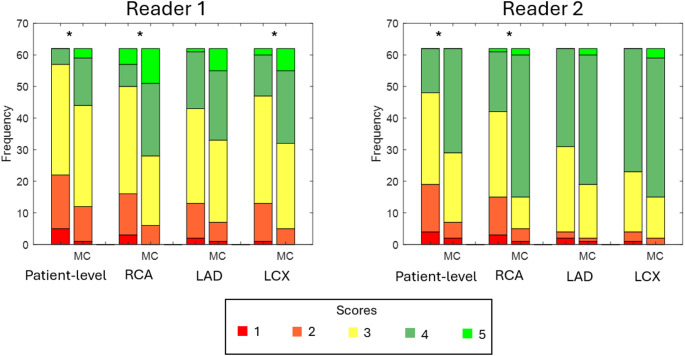
Score distribution of the two readers for uncorrected and motion-corrected images, for all patients included in the study. The asterisk denotes statistical significance. IQ = patient-level image quality; RCA = right coronary artery; LAD = left anterior descending artery; LCX = left circumflex artery; MC = motion-corrected

The radiologist image quality scoring results are shown for both readers in Fig. 4. On a per-patient basis, the number of cases scored 3 or higher on the uncorrected images was 40/62 (64.5%) and 43/62 (69.4%) respectively for reader 1 and 2. On the motion-corrected images, this number improved to 50/62 (80.6%) and 55/62 (88.7%), respectively for reader 1 and 2. On a per-vessel basis, the right coronary artery (RCA) followed the same trend, with improvements in scoring from uncorrected to motion-corrected images of 16.1% for both readers. Improvements in scoring for the left anterior descending (LAD) and left circumflex (LCX) arteries were more limited, between 3.3% and 9.7% (LAD) and 3.3% – 12.9% (LCX). The number of cases deemed diagnostically interpretable (score ≥ 2, patient-level) were 57/62 (91.9%) and 58/62 (93.5%) on the uncorrected images, and 61/62 (98.4%) and 60/62 (96.8%) on the motion-corrected images (respectively for reader 1 and 2).

With the application of the MC software, assigned scores improved at least one point in 41.9% and 48.4% of the cases on a per-patient basis (reader 1 and 2, respectively). No motion-corrected image was scored less than the respective uncorrected image for either reader.

For both readers, the difference in scores between uncorrected and motion-corrected images were found to be statistically significant for patient-level image quality (*p* < 1.5e-2) and for the RCA (*p* < 1.0e-4). No significance was found for the LAD, while significance on the LCX was achieved only by reader 1 (*p* = 0.04). In the subgroup analysis with cut-off value at 75 bpm, no significant difference was found in score distribution between the groups of patients with HR ≤ 75 bpm vs. HR > 75 bpm at neither patient or vessel level for either reader (*p* > 0.10).

No correlation was found between the differences in scoring from uncorrected to motion-corrected images and any patient or imaging characteristics (*p* ranging between 0.08 and 0.98), with the only exception of HR variability (range) for reader 2 (*p* < 0.01).

Interobserver agreement (Gwet) was found to be between [0.67–0.78]; all Gwet coefficients on a patient and vessel-level and shown in Table 2.

**Table 2:** Interobserver agreement. RCA = right coronary artery; LAD = left anterior descending artery; LCX = left circumflex artery

		Gwet agreement coefficient
Uncorrected	Patient-level	0.78
	RCA	0.78
	LAD	0.70
	LCX	0.67
Corrected	Patient-level	0.74
	RCA	0.73
	LAD	0.73
	LCX	0.75

Results of the objective image quality analysis are reported in Table 3. No significant differences in image noise, SNR and CNR were found between uncorrected and motion-corrected images (*p* = 0.62, *p* = 0.28, *p* = 0.93, respectively). The ERD was found to be significantly lower on the motion-corrected images (*p* = 0.01), and the ERS significantly higher on the motion-corrected images (*p* < 1.0e-3).

**Table 3:** Noise measurements. Data are mean (standard deviation)

Objective measurement	Uncorrected	Motion-corrected	*p*
Image noise, HU	20.86 (2.23)	20.84 (2.18)	0.62
SNR	21.00 (6.34)	21.03 (6.36)	0.28
CNR	24.39 (7.13)	24.41 (6.83)	0.93
ERD, mm	2.22 (0.50)	2.13 (0.49)	0.01
ERS, HU/mm	155.91 (78.65)	200.63 (83.99)	< 0.001

HU = Hounsfield units; SNR = signal-to-noise ratio; CNR = contrast-to-noise ratio; ERD = edge rise distance; ERS = edge rise slope

**Fig. 5:**
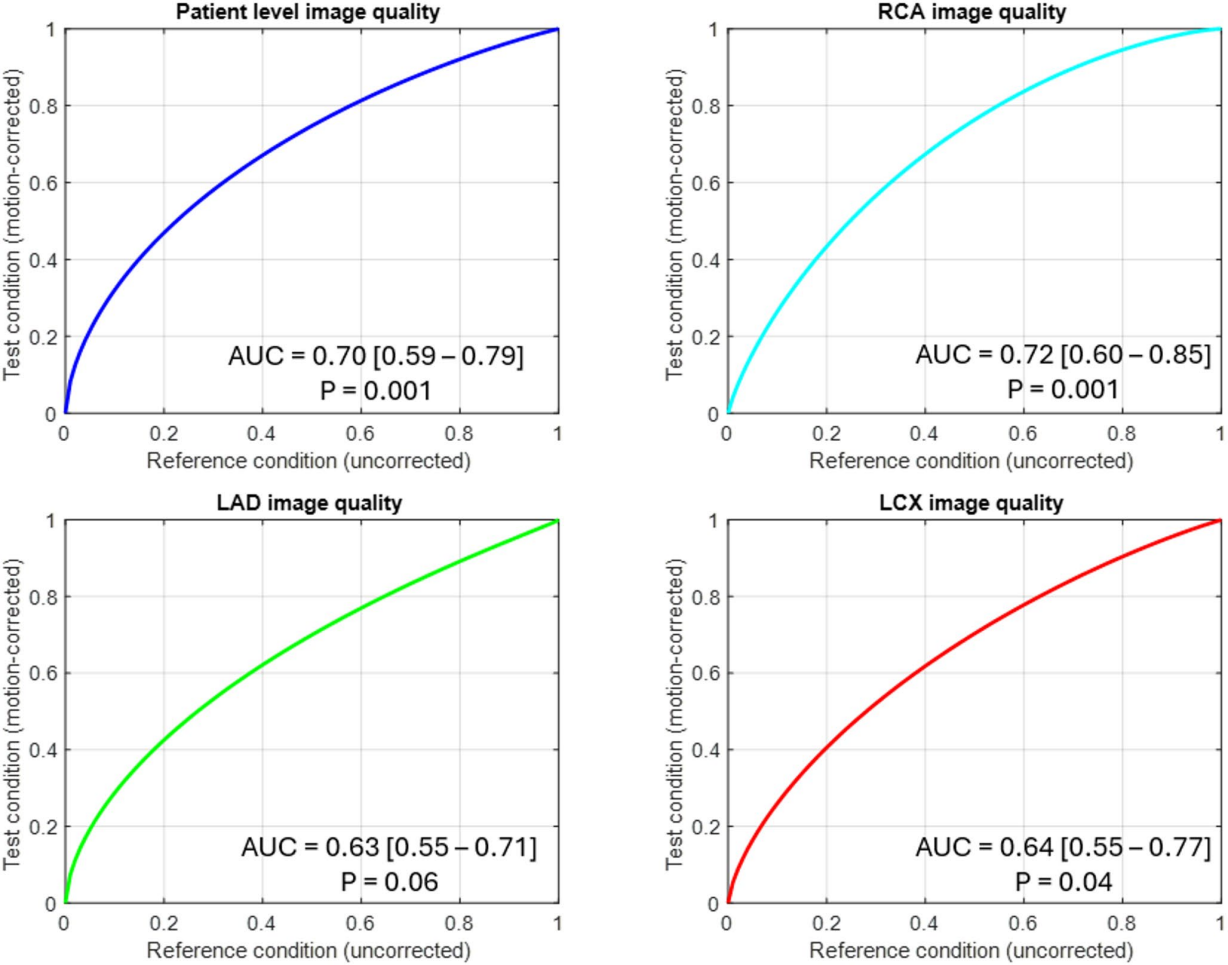
Results of the VGC analysis for all patients included in this study. Results are reported on a patient- and vessel-level. P values indicate significance difference in image quality scoring between uncorrected and motion-corrected images. RCA = right coronary artery; LAD = left anterior descending artery; LCX = left circumflex artery

Results of the VGC analysis (binormal VGC curve, random reader analysis, 2000 permutations, 2000 bootstraps) are reported in Fig. 5. Both patient level and RCA image quality scoring resulted in significant difference between uncorrected and motion corrected images (*p* < 0.01). A weak significance was found for the LCX (*p* = 0.04), while no significance was found for the LAD (*p* = 0.06), confirming the results of the previously described analysis performed per-reader.

**Fig. 6:**
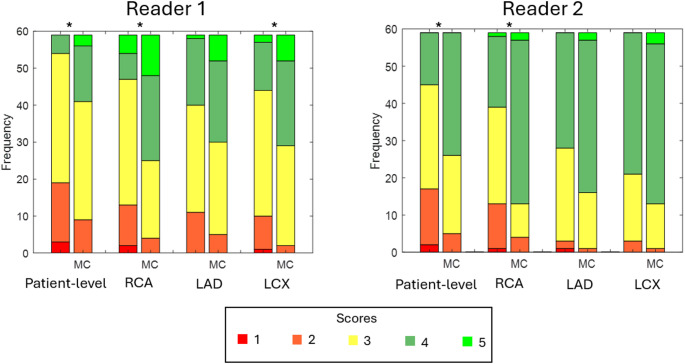
Score distribution of the two readers for uncorrected and motion-corrected images, after exclusion of the cases deemed non-interpretable due to reasons unrelated to MC performance (failed contrast timing, suboptimal acquisition window). The asterisk denotes statistical significance. IQ = patient-level image quality; RCA = right coronary artery; LAD = left anterior descending artery; LCX = left circumflex artery; MC = motion-corrected

Three out of the 62 cases (4.8%; HR at acquisition: 74 bpm, 75 bpm and 104 bpm) remained non-interpretable for at least one reader after the application of MC. In two patients, this was caused by failed contrast timing resulting in insufficient opacification of the coronary arteries. In the remaining patient, the HR rose unexpectedly from 55 bpm during the breath hold exercise to 104 bpm during acquisition, resulting in a suboptimal acquisition window. A sub-analysis of subjective image quality scoring excluding these cases (as they were deemed non-interpretable for reasons unrelated to motion correction performance) is reported in Fig. 6. After excluding these cases, no case was deemed uninterpretable after the application of motion correction, and no changes in statistical significance was observed for any of the analyses performed and described above.

**Fig. 7:**
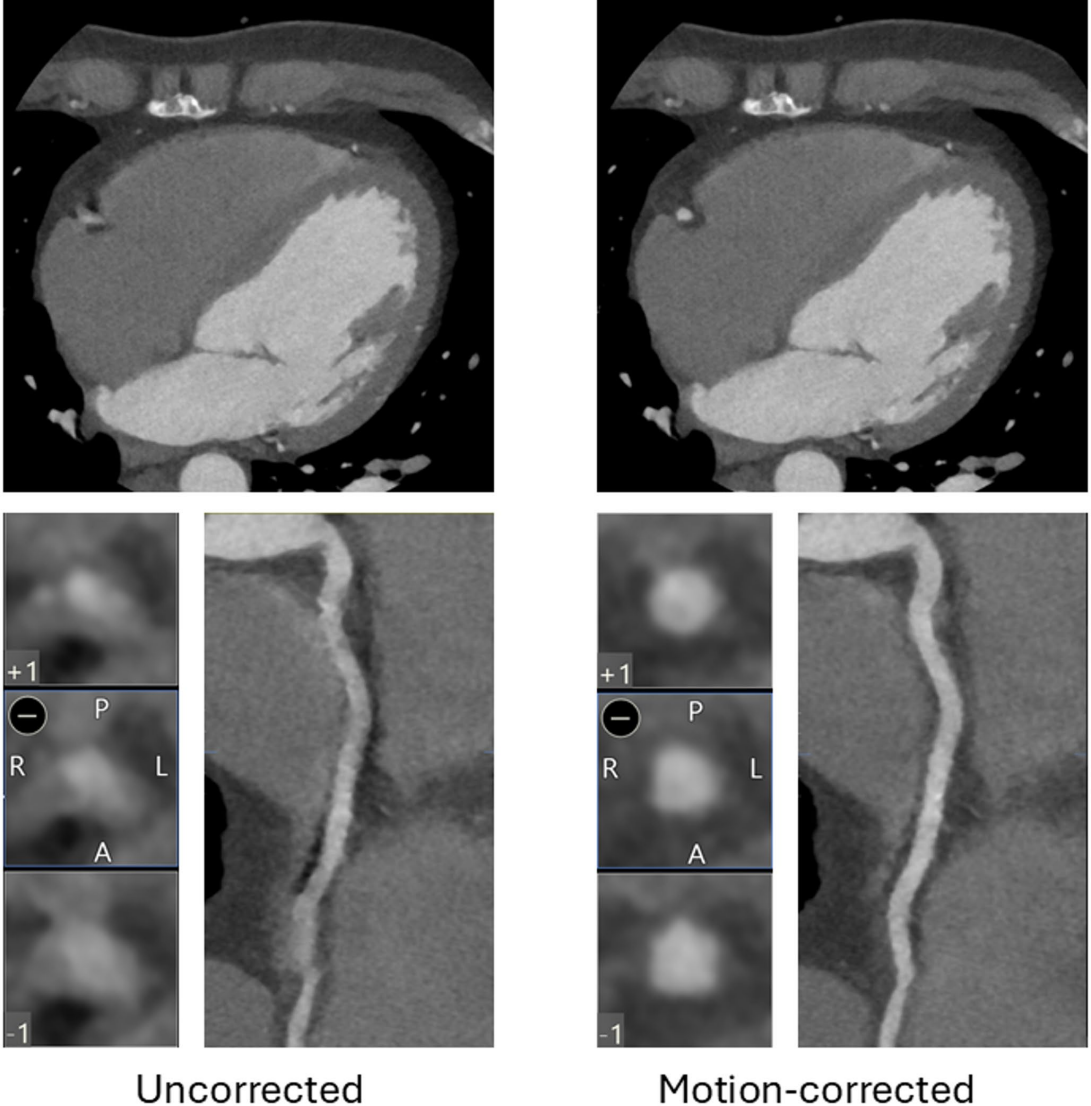
Uncorrected (left panel) and motion-corrected images (right panel) of a 36-year-old male patient (BMI, 22.63 kg/m^2^) with a mean heart rate at acquisition of 72 bpm. Cross-sectional images of the whole heart are shown in the top part, and three cross sections of the right coronary artery and curved multi-planar reconstruction are shown in the bottom part. The patient-level scores assigned to this case in the subjective image quality analysis were 2 (reader 1) and 3 (reader 2) in the uncorrected reconstruction, and 4 (both readers) in the motion-corrected reconstruction

**Fig. 8:**
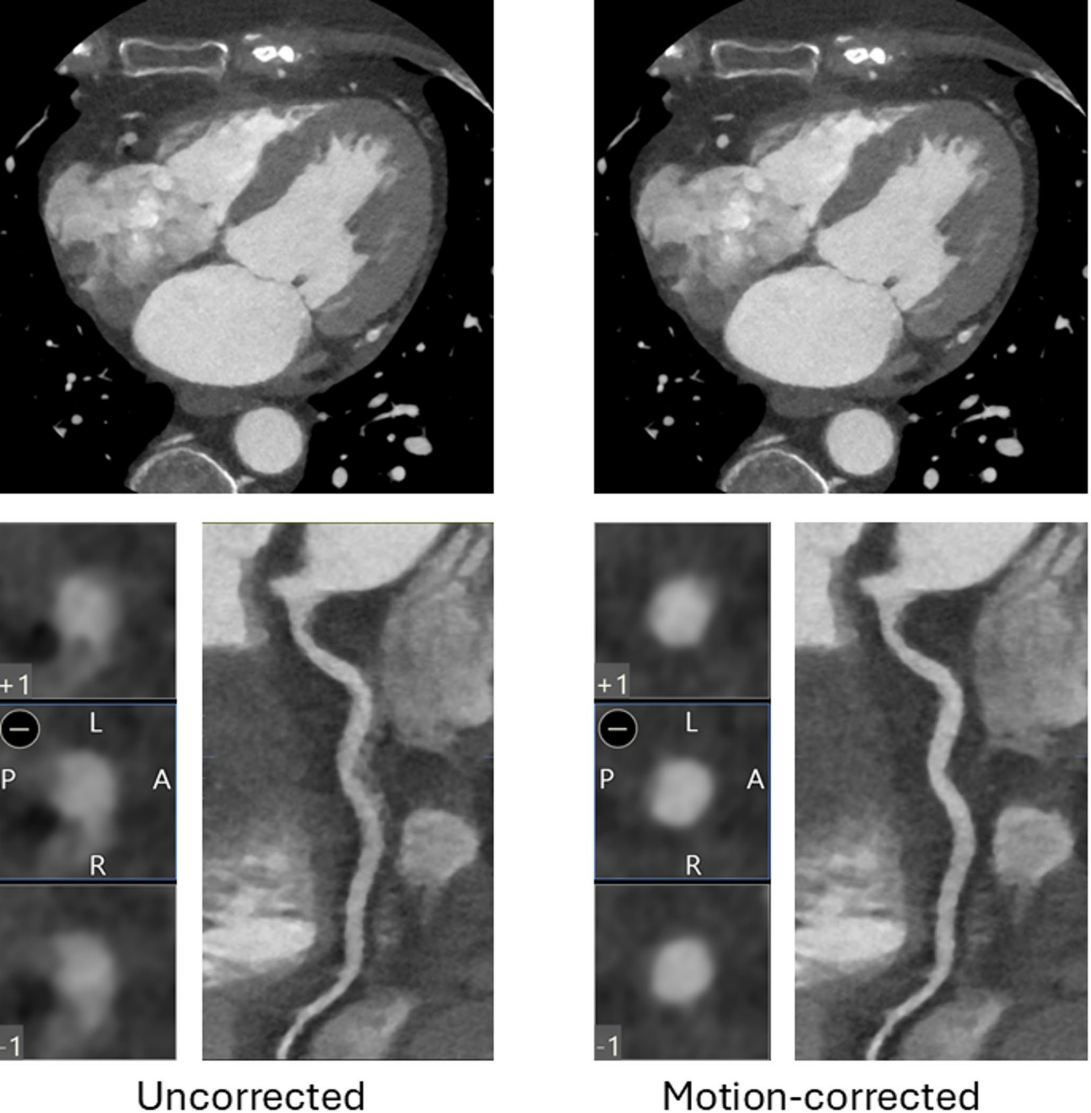
Uncorrected (left panel) and motion-corrected images (right panel) of a 70-year-old female patient (BMI, 24.09 kg/m^2^) with a mean heart rate at acquisition of 95 bpm. Cross-sectional images of the whole heart are shown in the top part, and three cross sections of the right coronary artery and curved multi-planar reconstruction are shown in the bottom part. The patient-level scores assigned to this case in the subjective image quality analysis were 3 (both readers) in the uncorrected reconstruction, and 4 (both readers) in the motion-corrected reconstruction

Two example cases (uncorrected and motion-corrected) are shown in Figs. 7 and 8.

## Discussion

The MC software evaluated in this study significantly reduced coronary artery motion artifacts and improved the fraction of diagnostically-interpretable cases in single-beat wide-area detector CCTA in patients with increased HR, in line with previous results obtained on simulated data [12]. Artifact reduction was found to be more pronounced on the RCA. A weakly-significant effect was found for the LCX (only one reader), and no significant effect was found for the LAD. While this may be due to the limited sample size, this is confirmed by previous literature that also reported a higher effect of motion correction algorithms on the RCA [4, 6], in line with clinical knowledge of the left arterial system being subjected to a substantially lower motion magnitude compared to the right system. The MC software did not change noise and contrast characteristics (compared to the uncorrected reconstructions), and the quantitative analysis of the ERD and ERS confirmed a reduction of artifacts in the motion-corrected images. The MC software never resulted in decreased scored image quality.

To the best of our knowledge, this is the first study, albeit of exploratory nature, to evaluate the potential of a DL PAR-based MC software for coronary artery motion correction in the normal clinical routine. The findings derived from this study with consecutive patients with increased HR therefore represent a step forward in understanding the application of DL PAR-based MC algorithms in CCTA.

The performance of the MC correction software evaluated in this study was not found to be correlated with any patient or imaging characteristics, with the exception of a mild significant correlation with HR variability for only one reader. Although these findings are in line with previous research on patients with increased HR [18], they likely reflect the limited statistical power deriving from exploratory cohorts and by a narrow HR distribution, rather than a true absence of relationship with biological of technological characteristics. Therefore, these findings should be further assessed in future studies with larger patient cohorts.

Previous findings on patients with increased HR obtained with MC approaches based on multi-phase reconstruction reported varying degrees of performance, with the fraction of cases deemed non-diagnostically interpretable after the application of MC ranging between 4.9% and 20.8% [8–10]. One of these studies was performed without HR control, after exclusion of patients with non-sinus rhythm, poor contrast timing or excessive noise, and inappropriate cardiac phase selection during acquisition [9]. The other two studies reported patients with an average lower patient BMI (24.8 kg/m^2^, 23.9 kg/m^2^) compared to that of the present study. Other findings, also focusing on patients with increased HR, reported a 16% improvement in coronary segment interpretability, with 14 out of 86 segments turning diagnostic [4]. Another previous study showed potential in phase-based motion correction approaches in 150 patients with a wide HR range, but showed no significance in improvements in subjective image quality when stratifying on the increased HR subgroup (≥ 70 bpm, 50 patients) [30]. These studies reported patient cohort sizes (with increased HR) ranging between 25 and 81 patients, therefore in line with the present study. Interobserver agreement between radiologists was found in line with previous studies [3, 8, 18, 24]. The exploratory findings of our study seem to point to an overall concordance with the results obtained in previously published work on phase-based approaches, suggesting at least non-inferiority in performance. However, this can only be interpreted as an overall qualitative comparison aimed to contextualize our work within the current panorama of scientific literature, and should not be interpreted as a direct result comparison, which is hampered by differences in methodology, inclusion and exclusion criteria, patient characteristics, CT scanner, and formal analysis. Additional and more accurate insights on any of such comparisons may only be achieved in the future by studies involving a substantially larger patient cohort possibly from different centers, additional readers, and consensus on methodological approach and formal analysis.

In this study, we focused on evaluating improvements in image quality and diagnostic interpretability deriving from the application of a new DL PAR-based MC software, as performed by previous studies evaluating phase-based MC approaches [8–10, 30]. This evaluation represents the essential first step in the assessment of a new MC algorithm, as it determines whether (and to what extent) it can support radiologists in clinical practice. Considering the findings of the present work, future studies will focus on the evaluation of the MC software on the impact on diagnostic measures, including its potential effect on small calcification detection and on plaque quantification.

A previous retrospective study evaluated the same DL PAR-based MC software described in this work for artifact reduction in the ascending aorta for the evaluation of aortic dissection [31]. Results are in line with our findings, pointing to an improved diagnostic interpretability on motion corrected images, and therefore confirm the potential of PAR-based methods on a different clinical application and on images acquired with different settings (contrast-enhanced chest CT protocol).

For patient inclusion, we chose a cut-off value of HR at acquisition of 70 bpm. While motion artifacts can be expected to be more prominent at higher HRs, previous studies including patients with HR lower than 75 bpm observed that motion can still be present, and therefore could still benefit from potential image quality improvements brought by MC algorithms [10, 18]. Furthermore, our choice of cut-off value at 70 bpm for patient inclusion was also in line with a recent study with 4085 patients, which shows presence of motion artifacts also in lower heart rates, and how the fraction of non-interpretable cases due to motion increases after 70 bpm [32].

No significant difference was found in magnitude of motion artifact correction between the subgroups of patients with cut-off threshold of 75 bpm. This suggests that the effect of motion correction seems to not be influenced by HR when this is above 70 bpm, at least for the exploratory cohort evaluated in this study, although this might be due to the limited sample size that hampers statistical significance in stratified analyses. This is in line with previous literature with similar cohorts and similar HR ranges [10, 18], where the strength of artifact correction was not found to be related with HR at acquisition. It can be expected, however, that this relationship could become more apparent if patients with lower HR (< 70 bpm) were included. For HRs above 70 bpm, future studies with larger cohorts are needed to reach a sample size large enough to appreciate potential differences in artifact reduction strength as a function of heart rate. These future studies should therefore expand on the findings reported in our work, which are to be considered preliminary and aimed to demonstrate clinical feasibility rather than establishing the conclusive superiority or necessity of the MC software in certain patient groups.

When performing image reconstruction, a super-resolution deep learning method with matrix size of 1024 × 1024 was used, as routinely performed at our institution for cardiac CT examinations and as recommended by the CT manufacturer. This is motivated by the fact that a larger matrix size coupled with deep learning reconstruction has been shown to reduce blooming artifacts and improve stent, plaque and calcification assessment [19–20]. With this matrix size, motion is expected to be amplified thanks to the improvement in image spatial resolution, and therefore this matrix size is expected to benefit the most from motion correction approaches, compared to the standard 512 × 512. However, as shown by previous studies with modern CT scanners, motion can still be present at elevated heart rates even in images reconstructed in standard matrix size [32], and thus motion correction algorithms can still play a key role in enhancing image interpretability even at standard resolution. The benefits of the MC software investigated in this study on images reconstructed with standard reconstruction methods and with a standard matrix size should be therefore evaluated in future work.

This study has limitations. First, it was performed in a single center. While this is commonly performed, results may be limited in generalizability and should therefore be confirmed in future multicenter studies. Second, the number of subjects included, although similar to previous studies [3, 9–10], was limited due to targeting only increased HR patients, and this might have caused some statistical tests to not reach significance (e.g., correlation with patient and imaging characteristics). Third, only one DL PAR-based MC software (CLEAR Motion) was evaluated in this study, and therefore results might not be translatable to other algorithms. Fourth, the analysis performed to evaluate the correlation between differences in image quality scoring (motion-corrected vs. uncorrected) and degree of coronary artery calcification was based on subjective scoring. As this was scored subjectively by an expert reader (due to the Agaston score not being routinely performed at our institution), it should be intended as a supplementary qualitative analysis and not as a substitution of the Agaston score as a standardized methodology. Another limitation is given by the number of included patients with very challenging motion conditions. While the evaluated cohort encompasses a HR range between 70 bpm and 123 bpm, only 40% of the patients presented HR at acquisition above 80 bpm. This is due to the use of beta blockers in our normal clinical routine for CCTA imaging, which did not allow for a comprehensive analysis in patients with serious motion conditions dictated by considerably high HRs. Although previously reported cohorts also present a similar (or lower) range of heart rates [8, 10, 18], this can limit the ability of our findings to comprehensively and conclusively understand the potential of the MC software in improving the fraction of diagnostically interpretable cases in very challenging patients that would be considered non-interpretable in the absence of motion correction. It should be noted, however, that recent literature evaluating 4085 patients highlighted that motion artifacts can still occur also at lower heart rates and also with modern CT technology [32], therefore justifying the cut-off value of inclusion of 70 bpm chosen in our study and the evaluation of the MC software in this cohort. Finally, the effect of the MC software on diagnostic accuracy relative to a reference standard (e.g., invasive coronary angiography) was not evaluated. This is in line with most previous studies on a similar subject [3, 4, 10, 18], and is motivated by the fact that non-interpretable CCTA images are to be considered as positive findings for disease. Therefore, it can be expected that the improved diagnostic interpretability brought by the MC software could improve diagnostic performance [33]. This should be confirmed in future studies that evaluate whether the improvements in image quality and interpretability found in the present work translate in any improvement in diagnostic accuracy or in any change in patients management (e.g., fraction of patients referred to re-scan or invasive imaging, also accounting for risk profile and calcium score).

In conclusion, and pending future verification in larger multi-center studies, the evaluated MC software showed high potential in correcting coronary artery motion artifacts in patients with increased HR in single-beat wide-area detector CCTA.

## Data Availability

The data underlying this article will be shared on reasonable request to the corresponding author.

## Abbreviations

MC: motion correction
DL: deep learning
PAR: partial angle reconstruction
HR: heart rate
CCTA: coronary computed tomography angiography
BMI: body mass index
MVF: motion vector field
CAD: coronary artery diseases
CTDI: computed tomography dose index
DLP: dose length product
ED: effective dose
ROI: region of interest
SNR: signal-to-noise ratio
CNR: contrast-to-noise ratio
SD: standard deviation
IQR: interquartile range
HU: Hounsfield units
p: P-value
RCA: right coronary artery
LAD: left anterior descending artery
LCX: left circumflex artery
VGC: visual grading characteristics
ROC: receiver operating characteristics
ERS: edge rise slope
ERD: edge rise distance
